# Association of Visual Defects and Occlusal Molar Class in Children

**DOI:** 10.1155/2018/7296289

**Published:** 2018-06-25

**Authors:** Silvia Caruso, Roberto Gatto, Mario Capogreco, Alessandro Nota

**Affiliations:** ^1^Department MeSVA, University of L'Aquila, L'Aquila, Italy; ^2^Dental School, Vita-Salute San Raffaele University, Milan, Italy

## Abstract

**Purpose:**

The purpose of this cross-sectional study is to evaluate the presence of any correlations between dysfunctions related to visual impairments and dental occlusion.

**Methods:**

The test group included 34 subjects (21 males and 13 females; mean age 11 ± 2 years) randomly selected with the following inclusion criteria: absence of any diagnosis for problems at visual level except those related to refractive defects, visual acuity of at least 1.0, absence of any syndrome or malformation in the craniofacial area, good general health, and absence of any systemic disease able to influence the vision or the craniofacial growth. They underwent visual clinical tests to evaluate the presence of fusional vergence defects and amplitude. Each patient underwent an orthodontic clinical exam and the occlusal molar relationship of each subject was recorded and considered as occlusal variable. A statistical analysis with Chi-Squared test was performed in order to analyze the associations between the visual defects and the occlusal variable.

**Results:**

A statistically significant association between the molar occlusal relationship and the occurrence of exodeviations was observed. The percentage of subjects presenting fusional amplitudes with convergence lower of the cut-off value was statistically significantly higher in the group of occlusal molar second class.

**Conclusions:**

The results obtained show that there is an association between occlusal second molar class and fusional vergence defects.

## 1. Introduction

Several embryological, neurofunctional, and anatomic correlations have been suggested between the stomatognathic and oculomotor systems; in fact, the oculomotor system has origins in the occipital somites as the tongue muscles and suboccipital muscles [1–4]. Furthermore, the trigeminal system represents the connection between the stomatognathic structures derived from the branchial arches and the somitic structures [5].

On these anatomical bases, some previous studies hypothesized a clinical association between visual defects and oral malocclusions [6–8]. Different studies [9–12] proved a clinical association among temporomandibular disorders, occlusal features, and the presence of ocular convergence defects. A significant input of visual proprioception on the electric activity of masticatory muscles was also assessed [8, 13]. Monaco et al. [14–17] found a different prevalence of visual defects among children with different orthodontic skeletal classes.

A clinical association was also enlightened by a clinical case report that showed a resolution of strabismus after treating a subject with transverse maxillary constriction with rapid maxillary expansion technique [1].

Fusional vergences are optomotor reflexes designed to maintain the alignment of the eyes so that similar retinal images project on corresponding retinal areas. The horizontal fusional vergences are further subdivided into fusional convergence and divergence. The fusional convergence controls the exophoria, thus eliminating the bitemporal retinal disparity, while the fusional divergence eliminates the binasal retinal disparity and controls the esophoria. These visual defects in pediatric age are considered latent forms of strabismus.

Currently the scientific literature lacks studies about the relationships between the dental occlusion and fusional vergences [5]. The existence of this kind of relationship could allow an early diagnosis of latent vergence problems in subjects with malocclusions.

The purpose of this cross-sectional study is to investigate the association between oral malocclusions and fusional vergence visual defects in a pediatric population.

## 2. Materials and Methods

The test group included 34 subjects (21 males and 13 females; mean age 11 ± 2 years) randomly selected among patients from the Dental Paediatric Clinic of the San Salvatore hospital of L'Aquila, Central Italy. Inclusion criteria were as follows: absence of any diagnosis for problems at visual level except those related to refractive defects, visual acuity of at least 1.0, absence of any syndrome or malformation in the craniofacial area, good general health, and absence of any systemic disease able to influence the vision or the craniofacial growth. The study was conducted in accordance with the Declaration of Helsinki between June 2016 and March 2017 and it was ethically approved by the institution.

All the subjects were enrolled in the study, after signing a proper consent form, with a random choice, disconnected from any criteria other than that of a minimum collaboration to carry out all the tests and an accurate physiological and pathological anamnesis of the patient was performed on each subject. The parents of each child were instructed about the modalities and purposes of this experimental study, and each test was previously explained in detail to the subjects involved in the study, according to their level of comprehension. In addition, each patient underwent a clinical dental examination in which, besides a generally orthodontic clinical exam, the occlusal molar relationship of each subject was recorded and considered as occlusal variable.

The performed visual clinical tests were aimed at evaluating the sensorial visual condition of each subject, recording as visual variables the following: (a) the presence of visual horizontal vergence defects classifying subjects as affected by orthophoria, exophoria, or esophoria and (b) the amplitude of fusional vergences classifying subjects as higher or lower of the related cut-off value.

The maximal amount of eye movement produced by fusional vergences is referred to as “amplitude of fusional vergences.” The amplitudes of horizontal fusional vergences are thus measured in prism diopters (Δ).

During the test, the clinician uses the horizontal prisms and records two specific points. The first point is when the patient stops to converge further and may recognize a diplopia: at that moment the examiner can see the patient's eyes break from the point of maximal convergence; this is called fusion break point. Then, the examiner reduces the prism power and decreases the disparity in the retinal image or reduces the convergence of the major amblyoscope tubes, until fusion is restored, and the target is seen clearly; this is called fusion restoration point.

The normal fusional convergence amplitude is set at a specific value of Δ for fusion break and at a lower level of Δ for fusion restoration; this range is usually considered the normal range.

### 2.1. Data Analysis

Descriptive statistics was preliminarily performed, as percentage distributions. Then, the association (cross-tabulation) between the visual and dental occlusion data (the presence/absence of an atypical swallowing and the occlusal molar relationship) was investigated. In the reading of the data, due to the small sample, the quantitative values have been preliminarily categorized in qualitative forms, assigning a “normal” value (cut-off value) to each category, and then dichotomized as follows: ≥ of the cut-off value or < of the cut-off value.

For the data related to the convergence point, the value of 5 cm was considered as the breaking point, whereas the cut-off value for the possibility of recovery was placed at 2.5 cm from the breaking point. Then, regarding the fusional amplitudes, a cut-off value of 20 Δ was fixed for the convergence and the value of 12 Δ for the divergence. Cross tabulations were constructed for the presence/absence of atypical swallowing, and for the occlusal molar relationship. Data in each of the groups were compared with the Chi-Square test, and the significance was set at 0.05.

The sample size was calculated a priori in order to achieve a sample power of 80% with an alpha error of 0.05 for the primary outcome and a minimum of 32 subjects was required.

## 3. Results

All subjects successfully completed the test, and the data were accurately collected.

### 3.1. Oculomotor Exam

Out of a total of 34 subjects analyzed: 5 subjects presented orthophoria (15%), 6 subjects esophoria (17%), and 23 subjects exophoria (68%) while 12 subjects had fusional amplitudes in convergence lower than the cut-off value (35%) and 22 subjects higher than the cut-off value (65%).

### 3.2. Intraoral Exam

Out of a total of 34 subjects analyzed: 7 subjects presented a molar first class (20%), 23 subjects a molar second class (68%), and 4 subjects a molar third class (12%).

### 3.3. Relationship between Visual Defects and Dental Malocclusions

There was not such a clear diversification of the considered visual defects, stratifying the sample in two groups as absence/presence of atypical swallowing.

From the cross-tabulation among the visual defects and the distribution of the occlusal molar relationships in the sample emerged a statistically significant association between the molar occlusal relationship and the occurrence of exodeviations (p < 0.05) (Table 1). In particular, the presence of exodeviations is higher in the second molar class subjects. The percentage of subjects presenting fusional amplitudes with convergence lower than the cut-off value was statistically significantly higher in the group of occlusal molar second class (Table 2).

## 4. Discussion

Previously in literature, it was hypothesized that facial skeletal growth patterns could determine an altered development of the ophthalmic area. In fact, previous studies found an association of refractive visual defects with specific occlusal molar classes [14–17] while another study found an association between ocular convergence and occlusal vertical malocclusions [18], but no data are reported on the relationship between fusional amplitudes and occlusal molar relationship in children.

To the authors' knowledge this the first study that aimed to investigate the possible relationship between fusional vergence defects and amplitudes and the occlusal molar relationship in a pediatric population. As the subjects of this study were recruited in a dental pediatric clinic, the prevalence of dental malocclusions and other parameters could not represent the absolute rates of the general population.

The horizontal fusional vergence exam showed a clearly higher prevalence of the exophoria (67%) in the analyzed sample.

When the association between the occlusal parameters and the visual defects was analyzed, a statistically significant association was found between molar class II occlusal relationship and the presence of exophoria. Furthermore, the molar class II occlusal relationship was significantly associated with fusional amplitudes in convergence lower than the cut-off value.

At the light of this evidence, the high percentage of molar class II occlusal relationship observed in this sample owing to the subjects recruitment in a pediatric dental clinic explains the consequently higher percentage of subjects with exophoria.

A recent study published by Bollero et al. [5], in disagreement with the present study, observed an absence of association between molar class alterations and ocular motility or convergence defects, but it should be observed that as a parameter only the presence/absence of any kind of ocular motility defects or convergence defects was analyzed, and not the single defect type. Monaco et al. [17] could also be considered in disagreement with this study as they found an absence of associations between strabismus and molar class malocclusions.

Binocular defects in children were previously associated with different clinical situations and functional pathologies. For example, children-athletes have higher performance for the fusional amplitude in convergence, with respect to a matched control group of nonathletes [19]. In addition, binocular deficits were also highly observed in dyslexic children and thus considered as a result of their phonological deficit of dyslexia [20].

The present findings suggest a clinical association between the occlusal second molar relationship and fusional vergence defects. The early diagnosis of vergence anomalies—before they became symptomatic—is crucial to prevent a potential occurrence of a strabismus. Without treatment, some of these anomalies may decompensate and become strabismus. The present findings flanked by the previous literature could suggest that children with orthodontic diagnosis for occlusal defects as second molar relationship must be carefully evaluated for an early diagnosis of visual defects, mostly those related to refractive errors, anomalous, and binocular function.

Limitations of the present study are the reduced sample and the impossibility of determining a cause-effect relationship in the observed association; further studies are needed to overcome these limitations.

## 5. Conclusions

The results obtained show that there is an association between occlusal second molar class and fusional vergence defects. Consequently, defects and dental malocclusions could be related to the occurrence of visual anomalies. The orthodontic diagnosis of specific dental malocclusions as the second molar class relationship in growing subjects should suggest an accurate orthoptic evaluation allowing an early diagnosis of visual defects. Further studies with larger samples should be performed to clarify the etiology of this kind of association.

## Figures and Tables

**Table 1:** Absolute frequencies and relative percentages of visual axes deviations within the occlusal molar class groups.

	Total (%)	Molar occlusal class I (%)	Molar occlusal class II (%)	Molar occlusal class III (%)	p value
Orthophoria	5 (14.7%)	2 (28.6%)	2 (8.7%)	1 (25%)	p < 0.05*∗*
Esodeviations	6 (17.6%)	2 (28.6%)	3 (13%)	1 (25%)	
Exodeviations	23 (67.7%)	3 (42.8%)	**18** **(78.3%)**	2 (50%)	

*∗*Chi-Square test; p < 0.05.

**Table 2:** Absolute frequencies and relative percentages of fusional amplitudes in convergence < or ≥ of the cut-off value within the occlusal molar class groups.

	Total (%)	Molar occlusal class I (%)	Molar occlusal class II (%)	Molar occlusal class III (%)	p value
fusional amplitudes in convergence < cut-off	12 (35.3%)	2 (16.7%)	**10** **(83.3%)**	0 (0)	p < 0.05*∗*
fusional amplitudes in convergence ≥ cut-off	22 (64.7%)	5 (22.7%)	13 (59.1%)	4 (18.2%)	

*∗*Chi-square test; p < 0.05.

## References

[1] Monaco A., Tepedino M., Sabetti L., Petrucci A., Sgolastra F. (2013). An adolescent treated with rapid maxillary expansion presenting with strabismus: A case report. *Journal of Medical Case Reports*.

[2] Hedge S. P., Dayanidhi V. K., Sriram (2015). Study of pattern of change in handwriting class characters with different grades of myopia. *Journal of Clinical and Diagnostic Research*.

[3] Ballanti F., Baldini A., Ranieri S., Nota A., Cozza P. (2017). Corrigendum to “Is there a correlation between nasal septum deviation and maxillary transversal deficiency? A retrospective study on prepubertal subjects” [Int. J. Pediatr. Otorhinolaryngol. 83 (April 2016) 109–112] (S0165587616000550) (10.1016/j.ijporl.2016.01.036). *International Journal of Pediatric Otorhinolaryngology*.

[4] Lin S. Y., White G. E. (1996). Mandibular position and head posture as a function of eye dominance. *Journal of Clinical Pediatric Dentistry*.

[5] Bollero P., Ricchiuti M. R., Laganà G., Di Fusco G., Lione R., Cozza P. (2017). Correlations between dental malocclusions, ocular motility, and convergence disorders: A cross-sectional study in growing subjects. *ORAL and Implantology*.

[6] Milani R. S., De Periere D. D., Micallef J.-P. (1998). Relationship between dental occlusion and visual focusing. *The Journal of Craniomandibular & Sleep Practice*.

[7] Marchili N., Ortu E., Pietropaoli D., Cattaneo R., Monaco A. (2016). Dental occlusion and ophthalmology: A literature review. *The Open Dentistry Journal *.

[8] Monaco A., Cattaneo R., Spadaro A., Giannoni M., Di Martino S., Gatto R. (2006). Visual input effect on EMG activity of masticatory and postural muscles in healthy and in myopic children.. *European journal of paediatric dentistry.*.

[9] Monaco A., Streni O., Marci M. C., Sabetti L., Giannoni M. (2003). Convergence defects in patients with temporomandibular disorders. *The Journal of Craniomandibular & Sleep Practice*.

[10] Cuccia A. M., Caradonna C. (2008). Binocular motility system and temporomandibular joint internal derangement: A study in adults. *American Journal of Orthodontics and Dentofacial Orthopedics*.

[11] Tecco S., Nota A., Caruso S. (2017). Temporomandibular clinical exploration in Italian adolescents. *Cranio: Journal of Craniomandibular Practice*.

[12] Baldini A., Nota A., Cozza P. (2015). The association between Occlusion Time and Temporomandibular Disorders. *Journal of Electromyography & Kinesiology*.

[13] Monaco A., Cattaneo R., Spadaro A., D'Andrea P., Marzo G., Gatto R. (2006). Ocular correction effects on EMG activity of stomatognathic muscles in children with functional mandibular lateral- deviation: a case control study.. *European Journal of Paediatric Dentistry*.

[14] Monaco A., Sgolastra F., Petrucci A., Ciarrocchi I., D'Andrea P. D., Necozione S. (2013). Prevalence of vision problems in a hospital-based pediatric population with malocclusion. *Journal of Pediatric Dentistry*.

[15] Monaco A., Sgolastra F., Cattaneo R. (2012). Prevalence of myopia in a population with malocclusions. *European Journal of Paediatric Dentistry*.

[16] Monaco A., Spadaro A., Sgolastra F., Petrucci A., D'Andrea P. D., Gatto R. (2011). Prevalence of astigmatism in a paediatric population with malocclusions. *European Journal of Paediatric Dentistry*.

[17] Monaco A., Spadaro A., Sgolastra F., Petrucci A., D'Andrea P. D., Gatto R. (2011). Prevalence of hyperopia and strabismus in a paediatric population with malocclusions. *European Journal of Paediatric Dentistry*.

[18] Silvestrini-Biavati A., Migliorati M., Demarziani E. (2013). Clinical association between teeth malocclusions, Wrong posture and ocular convergence disorders: An epidemiological investigation on primary school children. *BMC Pediatrics*.

[19] Omar R., Kuan Y. M., Zuhairi N. A., Manan F. A., Knight V. F. (2017). Visual efficiency among teenaged athletes and non-athletes. *International Journal of Ophthalmology*.

[20] Wahlberg-Ramsay M., Nordström M., Salkic J., Brautaset R. (2012). Evaluation of aspects of binocular vision in children with dyslexia. *Strabismus*.

